# Finding and Not Finding Rat Perirhinal Neuronal Responses to Novelty

**DOI:** 10.1002/hipo.22584

**Published:** 2016-04-18

**Authors:** Eva von Linstow Roloff, Robert U. Muller, Malcolm W. Brown

**Affiliations:** ^1^ School of Physiology and Pharmacology, Medical Sciences Building, University of BristolUnited Kingdom; ^2^ Department of Physiology and PharmacologySUNY Downstate Medical Center, Brooklyn, New York. In memoriam, Robert U. Muller (1942–2013)

**Keywords:** recognition memory, medial temporal lobe, familiarity, electrophysiology, visual responses

## Abstract

There is much evidence that the perirhinal cortex of both rats and monkeys is important for judging the relative familiarity of visual stimuli. In monkeys many studies have found that a proportion of perirhinal neurons respond more to novel than familiar stimuli. There are fewer studies of perirhinal neuronal responses in rats, and those studies based on exploration of objects, have raised into question the encoding of stimulus familiarity by rat perirhinal neurons. For this reason, recordings of single neuronal activity were made from the perirhinal cortex of rats so as to compare responsiveness to novel and familiar stimuli in two different behavioral situations. The first situation was based upon that used in “paired viewing” experiments that have established rat perirhinal differences in immediate early gene expression for novel and familiar visual stimuli displayed on computer monitors. The second situation was similar to that used in the spontaneous object recognition test that has been widely used to establish the involvement of rat perirhinal cortex in familiarity discrimination. In the first condition 30 (25%) of 120 perirhinal neurons were visually responsive; of these responsive neurons 19 (63%) responded significantly differently to novel and familiar stimuli. In the second condition eight (53%) of 15 perirhinal neurons changed activity significantly in the vicinity of objects (had “object fields”); however, for none (0%) of these was there a significant activity change related to the familiarity of an object, an incidence significantly lower than for the first condition. Possible reasons for the difference are discussed. It is argued that the failure to find recognition‐related neuronal responses while exploring objects is related to its detectability by the measures used, rather than the absence of all such signals in perirhinal cortex. Indeed, as shown by the results, such signals are found when a different methodology is used. © 2016 The Authors Hippocampus Published by Wiley Periodicals, Inc.

## INTRODUCTION

There is a wealth of evidence that perirhinal cortex plays an essential role in recognition memory processes in humans, monkeys, and rats (see for reviews, Brown et al., 1987; Brown and Aggleton, 2001; Eichenbaum et al., 2007; Winters et al., 2008; Brown et al., 2010; Ranganath and Ritchey, 2012; Clark and Squire, 2013; Brown and Banks, 2015). For the monkey there has long been detailed data concerning how the responses of perirhinal neurons change with changes in stimulus familiarity (Brown et al., 1987; Riches et al., 1991; Eskandar et al., 1992; Fahy et al., 1993; Li et al., 1993; Miller et al., 1993; Miller and Desimone, 1994; Sobotka and Ringo, 1996; Xiang and Brown, 1998); see for reviews (Ringo, 1996; Brown and Xiang, 1998; Brown and Banks, 2015). In contrast, for the rat there have been few comparable studies of perirhinal neuronal response changes related to changes in stimulus familiarity (Zhu and Brown, 1995; Zhu et al., 1995; Young et al., 1997), although greater Fos expression for novel than familiar stimuli has been repeatedly found (e.g., Zhu and Brown, 1995; Zhu et al., 1996; Wan et al., 1999; Warburton et al., 2003; Warburton et al., 2005; Aggleton et al., 2012). However, more recent studies using an approach similar to that used in studying hippocampal place fields (Muller, 1996) have failed to find convincing evidence of perirhinal neuronal activity changes related to stimulus familiarity when rats explored a circular track containing novel and familiar objects (Burke et al., 2012; Deshmukh et al., 2012). The experiments reported here aimed to explore further the inconsistency between these findings by investigating perirhinal neuronal responsiveness in two different situations.

Accordingly, recordings of rat perirhinal neuronal activity were made in two situations related to those that have previously been used repeatedly to study the involvement of rat perirhinal cortex in recognition memory: the novel object preference test (Ennaceur and Delacour, 1988; Brown et al., 2012) and the paired viewing procedure (Zhu et al., 1996; Aggleton et al., 2012). In the novel object preference test, a rat demonstrates recognition memory by exploring a novel object more than an object that has previously been explored; this object recognition test has been used extensively to test perirhinal involvement in recognition memory (Barker and Warburton, 2011; Brown et al., 2012). In the paired viewing procedure a series of novel pictures are viewed with one eye, while the other eye sees a series of previously viewed pictures; this procedure has been used to demonstrate differences in activation between the perirhinal cortices in the left and right hemispheres, particularly for Fos (Zhu et al., 1996; Wan et al., 1999; Warburton et al., 2003; Warburton et al., 2005; Aggleton et al., 2012). In this experiments, a modified “paired viewing” procedure was used in which novel or familiar pictures were viewed with both eyes but other parts of the standard protocol were followed. Perirhinal neuronal responses related to stimulus familiarity were found using the modified paired viewing procedure but not using the object recognition test.

## MATERIAL AND METHODS

### Subjects

The subjects were five male Dark Agouti (DA: B&K, UK; Experiment 1) and two male Long Evans (Harlan, UK; Experiment 2) rats. Testing was done in accordance with the United Kingdom Animals (Scientific Procedures) Act of 1986 and had ethical approval from the University of Bristol Research Ethics Committee. Rats weighed 230–290 g at the time of surgery. They were housed on a reverse 12 h light/dark cycle (lights on at 6:00 pm). The rats had access to food and water for ≥2 h following each test session and for 48 h at weekends.

### Surgery

Each rat was anaesthetised with isoflourane and placed in a stereotaxic frame on a heating pad in preparation for attachment of a recording microdrive. Microdrives were an adaptation of the 4‐tetrode drive developed by Muller and Kubie (Kubie, 1984; Muller et al., 1987). Guide tubes (34 gauge, Coopers Needleworks, UK) protected the tetrodes and extended ∼5 mm from the bottom of the drive. The tetrodes extended ∼3 mm beyond the guide tubes. To reduce tissue damage, the tetrodes were created from straight (not twisted) 25 µm diameter 90% platinium‐10% iridium wires insulated with a H‐ML resin coat (Finewire, CAA) and the tetrode tips were cut at a 45° angle. The drive was implanted at the following coordinates from bregma: AP: −5.8 mm, L: 4.0 mm. The guide tubes were inserted at a 25° angle and lowered to 7.0 mm from the brain surface. Five screws provided attachment points to the skull and one served as ground for EEG recordings. Following attachment of the drive the exposed brain was covered with gelatin sponge (Spongostan, Johnson & Johnson Medical, UK). The drive was then embedded in bone cement containing gentamycin (CMW 1, de Puy, UK), anchoring it to the skull screws. Buprenorphine hydrochloride was administered i.m. for postoperative analgesia and Enroflaxin antibiotic was given s.c. prophylactically. The rats were left one week to recover before behavioral training commenced.

### Electrophysiological Recording

Electrophysiological recordings were made using a Cheetah recording system (Neurolynx, AZ). Animals were connected to the system via a custom built headstage, where signals from the individual leads of the tetrodes were passed through a low noise unity gain field effect transistor preamplifier, insulated multiwire cables and a commutator (Dragonfly, WV) to a distribution panel. Here, each of the 16 channels was amplified 10,000–40,000×, band‐pass filtered between 300 and 6000 Hz, and digitised at 30 kHz. Each time a signal on any of the four wires of a tetrode exceeded a set threshold of 50–100 μV an event was registered. All data were stored on a Windows XP station PC (Viglen, UK). Two wires from two different tetrodes were chosen as reference electrodes.

After recovery from surgery, the tetrodes were slowly advanced and multineuronal tetrode recordings sought several times a week until a stable population of cells was present. Single cells were then isolated offline using Spike Sorter (Plexon, TX; Gray et al., 1995). Only units with >150 spikes during a recording session were included in the analysis.

### Recording Situations

#### Experiment 1: “paired viewing” arena

**Figure 1:**
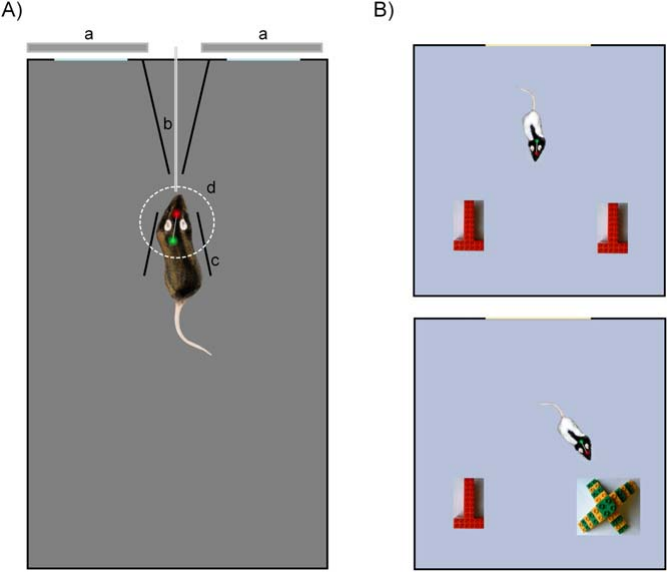
A: Diagrammatic representation of “paired viewing”‐like behavioral apparatus with rat in target position for picture presentation. Note the two LEDs on the headstage, allowing monitoring of position and head direction. “a”: monitor screens for image display; “b”: juice reward delivery tube; “c”: guide barrier; “d”: software‐defined target zone. B: Diagrammatic representation of object recognition arena: object configurations in Phase 1 (above) and Phase 2 (below). [Color figure can be viewed in the online issue, which is available at wileyonlinelibrary.com.]

Behavioral training and recording took place in a rectangular arena (45 × 80 × 60 cm) made from Perspex, centred in a 2.0 × 2.5 m dimly lit room and surrounded by a black circular curtain (∼1.2 m in diameter); Figure 1A. The viewing conditions were similar to those used in the paired viewing task that has demonstrated differences in Fos expression produced by viewing novel and familiar images (Wan et al., 1999), except that the same image was viewed by both eyes. Two identical images (10 × 11 cm) were projected on to two LCD screens 25 cm apart on one of the short sides of the arena. A central V‐shaped divider held a reward delivery tube ∼3 cm above floor level at a distance of 30 cm from the display screens. Two small barriers mounted in front of the reward tube helped the rat to locate itself in the correct position in front of the reward tube facing the projection screens.

An overhead camera (Fire‐I 501c, Unibrain, CA) and online motion tracking program (IC Imaging Control SDK, The Imaging Source Europe GmbH, DE) monitored the position and head direction of the rat in the arena according to a red (front) and green (rear) bright LED attached to the headstage (previously described by Huxter et al., 2007). Custom written online software (Dr. Yu Li, University of Manchester, UK) initiated picture presentations (2.0 s duration) when the rat had been correctly positioned facing the screens and the target zone in front of the reward tube for 1–2 s. Activation of a solenoid (which produced an audible click) 500 ms before the end of image presentation resulted in delivery of a drop of juice through the reward tube. A randomly variable intertrial interval (minimum 3.5 s) followed. Stimulus onset, stimulus offset and reward delivery markers were output (via a TTL box, USB‐6501, National Instruments, TX) to the Neuralynx recording software. If the rat left the target zone during stimulus presentation, the trial was marked invalid in the event record and no reward was delivered. Across days, rats were trained to correctly position themselves for viewing and then to view a set of 20 familiar images that were shown repeatedly in different pseudo‐random orders. Subsequently, recordings were made during the viewing of 80–120 pictures that included both highly familiar (viewed many times prior to the recording session) and novel (never previously seen) images with each image being repeated on the next trial.

##### Data analysis

The timestamps of action potential occurrence from the different isolated units were integrated with the event record by a custom built program (Dr Yu Li, University of Manchester, UK) that excluded data from invalid trials and sorted the timestamps according to picture category (first or second presentations of novel or familiar stimuli). NeuroExplorer (Neuralynx, AZ) then generated raster traces and peristimulus time histograms (PSTHs) of neuronal firing for each cell according to picture category. To analyse responsiveness, the 2 s before stimulus onset were used to establish a cell's baseline activity, the 2 s during stimulus presentation to establish any stimulus‐related activity, and the 2 s after the fluid delivery signal to assess reward delivery related activity. For each neuron, the mean spike count for the eight 250 ms bins in the 2 s before stimulus onset was compared with the counts in 250 ms bins for 2 s after stimulus onset for each trial and the significance of changes across all trials and all bins established by ANOVA (*P* = 0.05). Where there was overall significance, the significance of changes for individual bins was then established using the Bonferroni correction. The significance of any difference in response across different types of trials was established by repeated measures ANOVA (*P* = 0.05, Bonferroni‐corrected for multiple response types: novelty, familiarity, recency) with the factors stimulus type (novel or familiar), repetition (first or second appearance) and bin number.

#### Experiment 2: object recognition arena

Training and recording for the object recognition task took place in a 85 × 85 × 72 cm square arena, surrounded by circular black curtain in a room with dimmed light as for Experiment 1; Figure 1B. The object recognition task consisted of two phases separated by a 20 min delay. In Phase 1 (acquisition) two identical copies of a novel object (A) constructed from DUPLO (Lego, UK) were positioned at one end of the arena equidistant from the two corners, as in previous work (Barker and Warburton, 2011; Brown et al., 2012). Between trials the rat was placed in a dark container. In Phase 2 (choice/test), rats were exposed to another copy of the object A (now familiar) presented in Phase 1 and a different, novel object (B). The size of object A was 10 × 16 × 8 cm and object B 19 × 19 × 12 cm (w × l × h). In each phase the rat was released into the arena and allowed to explore it freely for a total recording time of 12 min. The first 4 min of this recording time was without disturbance to the rat's behavior, but thereafter food pellets were dropped randomly at a rate of 4 pellets/min to ensure the animals explored all of the arena.

To ensure that visually responsive neurones were present during object recognition testing, the population of cells was prescreened for visual responsiveness. While a rat moved freely within the empty arena, pictures were projected on to a white screen located on one end wall. Picture presentations (2 s duration) were followed by reward (a pellet dropped from a feeder above arena ∼3–6 s after picture onset). Picture presentations were initiated when the rat was facing the screen (at a minimum 20 cm distance), but were without other behavioral requirements. When visually responsive cells were found, the rat proceeded to object recognition testing and recording. Timestamps of the action potentials were registered as for Experiment 1. These times were related to the rat's position in the arena as recorded by the motion tracking program (Zynyuk et al., 2012), to allow the production of positional rate maps using NeuroExplorer (Neuralynx, AZ).

##### Data analysis

To analyse responsiveness to the objects and their familiarity, the arena was subdivided into a 4 × 4 grid of equal area squares. The firing for each cell was calculated when the rat was located within the different squares for each session. The firing rate in each square was then normalised to the overall firing rate across all 16 squares. The locations of squares that had activity levels >2 SD from baseline firing were noted. Activity levels within the 4 squares surrounding an object were compared with activity in squares surrounding other objects or activity distant from all objects (8 squares in the empty part of arena), within or between sessions. The statistical significance of activity differences between groups of squares was established using t‐tests (*P* = 0.05).

## RESULTS

**Figure 2:**
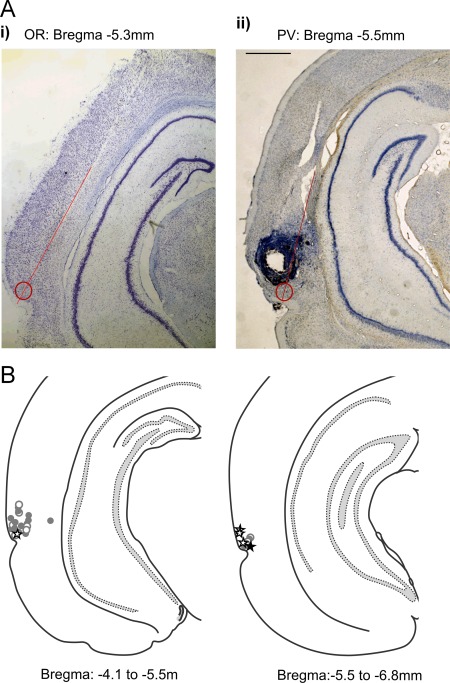
A: Histological sections of example recording locations (marked by a circle at end of line indicating tetrode track) where (i) an object‐related change in activity was recorded in the object arena and (ii) a recognition‐related response was recorded in the paired‐viewing arena. Recording locations were calculated from electrolytic lesions (see Aii) made through one of the implanted tetrodes at the end of all recordings. B: Diagrammatic representation on example sections of recording locations where task‐related activity was recorded. (NB multiple neurons were recorded at each site.) Neurons recorded between 4.1–5.5 mm and 5.5–6.8 mm behind bregma in either hemisphere have been projected onto sections of 5.0 and 6.0 mm behind bregma (Paxinos and Watson, 2005) Open circle: site with task‐related activity; filled circle: site also had recognition‐related activity; open star: site with object related activity; filled star: site also with recognition‐related activity during prescreening. [Color figure can be viewed in the online issue, which is available at wileyonlinelibrary.com.]

The positions of tetrodes were verified histologically after recordings terminated. Data are reported from recording sites judged to be in perirhinal cortex (Shi and Cassell, 1999) or posterior perirhinal cortex as defined by (Burwell, 2001) in areas 35 or 36 between 4.1 and 6.8 mm behind bregma (Paxinos and Watson, 2005); Figure 2. There was overlap in the locations of sites with task‐related responses in the two experiments.

### Experiment 1: Recordings in “Paired Viewing” Arena

**Figure 3:**
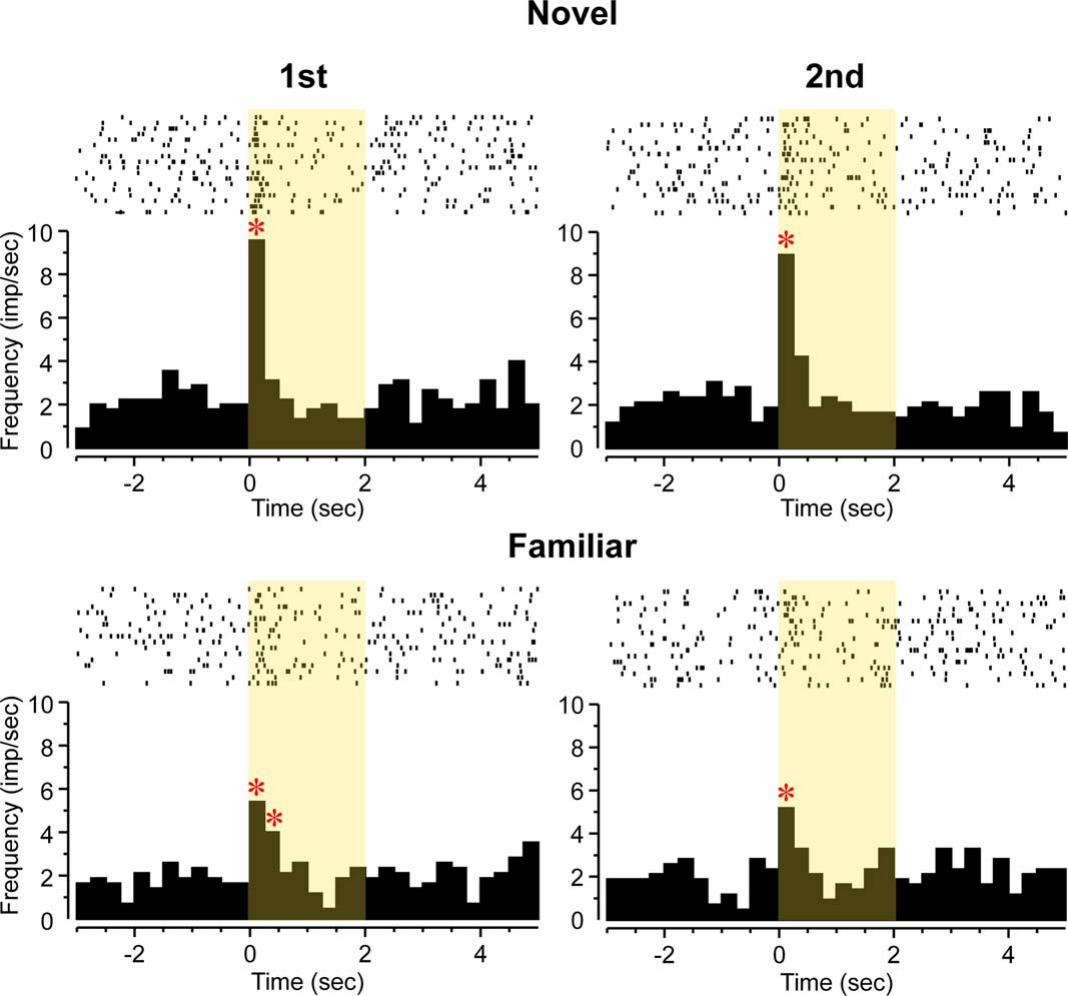
Perirhinal neuron whose activity increased (ANOVA: *F*_7,527_ = 20.87, *P* < 0.001) in relation to visual stimulus onset: “Familiarity” type response. Rasters of action potential occurrences are shown above cumulated PSTHs for four types of stimulus trials: first or second presentations of novel (N, upper row) or highly familiar (F; lower row) pictures. The neuron responded briefly but strongly to first and second presentations of novel stimuli but significantly less vigorously to first and second presentations of highly familiar stimuli (ANOVA, stimulus type, novel or familiar trials by bin: *F*_7,527_ = 3.35, *P* < 0.002). * Bin with activity significantly different (*P* < 0.05 after Bonferroni correction for multiple bins) from baseline (in this and subsequent figures). [Color figure can be viewed in the online issue, which is available at wileyonlinelibrary.com.]

**Figure 4:**
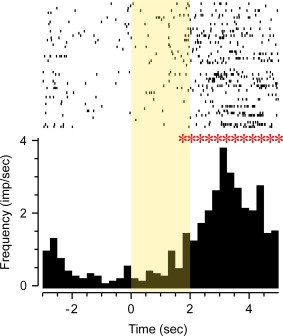
Perirhinal neuron with significantly increased activity (ANOVA: *F*_7,408_ = 3.45, *P* = 0.001) in relation to the timing of reward delivery (rasters of action potential occurrences above cumulated PSTH). A solenoid which produced an audible click was activated 1.5 s after the visual stimulus onset (time 0) and 0.5 s before stimulus offset; it led to the availability of a drop of juice from the juice reward delivery tube. [Color figure can be viewed in the online issue, which is available at wileyonlinelibrary.com.]

Activity was recorded within perirhinal cortex from a total of 120 well‐isolated single neurons during the viewing of images in a “paired viewing” arena (a rectangular arena on one wall of which images were displayed when the rat was facing it). A drop of juice was delivered just before the end of the display of each image. Of the 120 neurons, 30 (25%) changed their firing significantly subsequent to the onset of the visual stimuli. For 53% (16/30) of these neurons the activity change was an increase (Fig. 3). For 67% (20/30) of the neurons the response began within the first 250 ms following stimulus onset and did not last longer than 500 ms (Fig. 3). For the remainder, the activity change was gradual in onset (not significant in first 250 ms) but more prolonged. For 10% (3/30) neurons activity changed in the 2 s following the juice delivery signal (that included an audible click) rather than the onset of the visual stimuli (Fig. 4).

**Figure 5:**
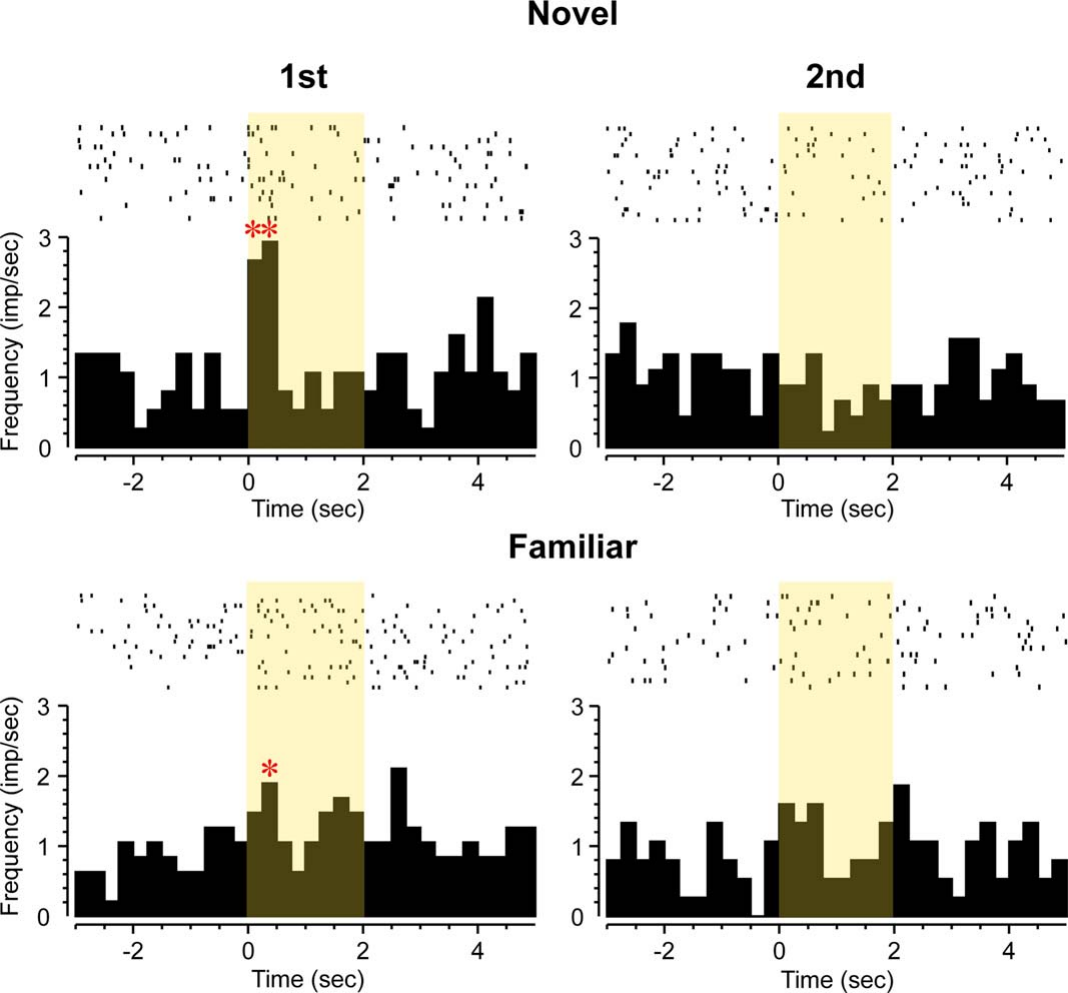
Perirhinal neuron with “novelty” type response. Rasters of action potential occurrences are shown above cumulated PSTHs for four types of stimulus trials: first or second presentations of novel (N, upper row) or highly familiar (F; lower row) pictures. Overall significant increase in firing (ANOVA: bin: *F*_7,511_ = 2.38, *P* = 0.02). The neuron responded significantly more to first but not second presentations of novel stimuli and not to first or second presentations of familiar stimuli (ANOVA, interaction between stimulus type [novel or familiar] and repetition [first or second trials]: *F*_1,511_ = 6.29, *P* = 0.01). [Color figure can be viewed in the online issue, which is available at wileyonlinelibrary.com.]

**Figure 6:**
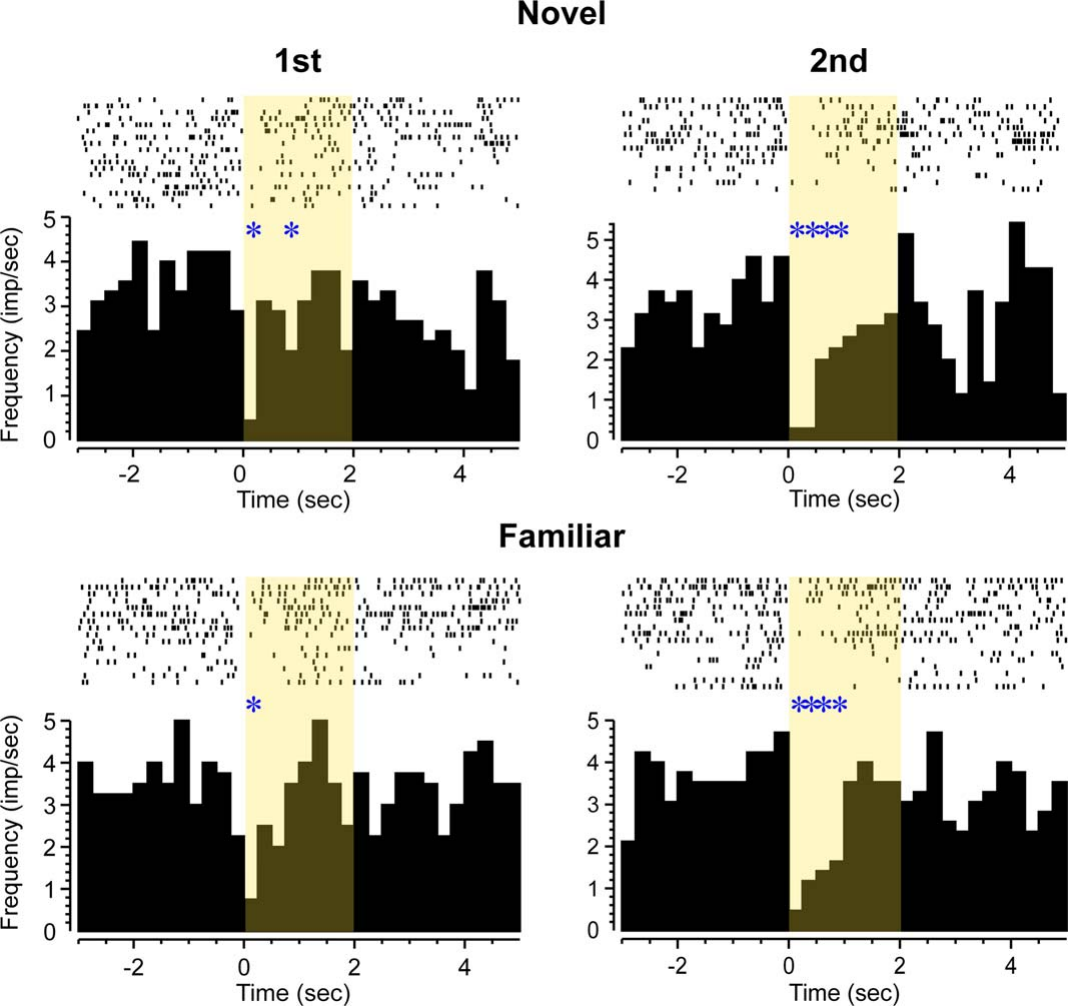
Perirhinal neuron with inhibitory “recency” type response. Rasters of action potential occurrences are shown above cumulated PSTHs for four types of stimulus trials: first or second presentations of novel (N, upper row) or highly familiar (F; lower row) pictures. The neuron's activity was strongly reduced after stimulus onset (ANOVA: *F*_7,495_ = 6.17, *P* < 0.001), significantly more so for the second presentations of novel or familiar stimuli than for their first presentations (ANOVA, repetition [first or second trials]: *F*_1,495_ = 4.16, *P* < 0.04). [Color figure can be viewed in the online issue, which is available at wileyonlinelibrary.com.]

The response of 19 neurons (63% of the 30 task‐related neurons; 16% of the total recorded), changed significantly dependent on the relative familiarity of the visual stimuli viewed. Such responses were found in four of the five rats from which recordings were made and their incidence did not very significantly across the rats (*χ* ^2^ test, *P* > 0.1). For 14 (73%) of these repetition‐sensitive responses, the change was a reduction in response for more familiar compared with less familiar stimuli. Examples are shown in Figures 3, 5, and 6. As previously described (Xiang and Brown, 1998) for monkey neuronal response changes on stimulus repetition, the types of change could be subcategorised. For 4 “novelty”' neurons, the response to novel stimuli differed significantly both to that when such initially novel stimuli were repeated and to that when highly familiar stimuli were presented (Fig. 5). For 6 “recency” neurons, the response changed significantly from the first to the second presentation either of novel or of highly familiar stimuli which had not been seen for at least a day (Fig. 6). For 6 “familiarity” neurons, the response to both the first and second presentations of an initially novel stimulus differed significantly from that to the first and second presentations of highly familiar stimuli (Fig. 3). The responses of the remaining 3 (16%) neurons changed significantly with stimulus familiarity but failed to conform to one of the above three patterns.

### Experiment 2: Recordings in Object Recognition Arena

Recordings were made from 15 perirhinal neurons while rats explored objects placed in a square arena. The recorded neuronal population had been prescreened for visual responsiveness (see Methods). In Phase 1 (acquisition) of the task, the object recognition arena contained two identical copies of a novel object (A); in Phase 2 (choice) the arena contained a third copy of the now familiar object (A) and a new novel object (B). For analysis of the recorded activity, the arena was subdivided into 16 equal squares; eight were adjacent to one or other of the two objects and eight were not. Activity was normalised to the mean firing rate across all 16 squares. The activity was first analysed for the presence of a change in any square that deviated by more than 2SD from the overall mean. The incidence of such changes was random in relation to the position of the objects.

**Figure 7:**
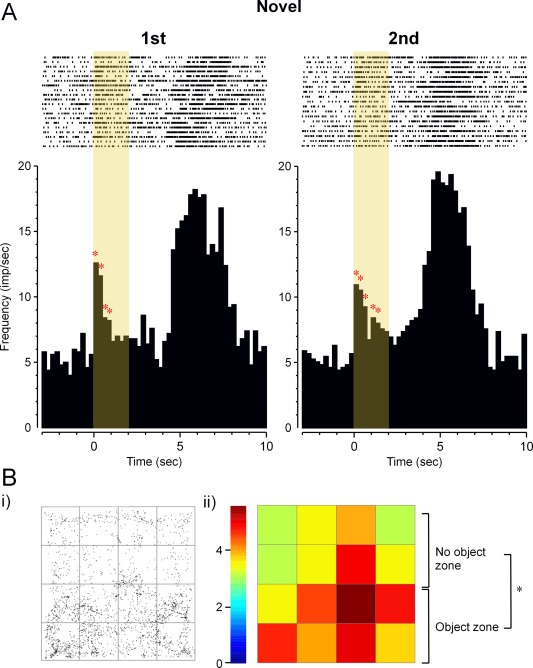
Perirhinal neuron's responses to pictures displayed during screening prior to recording in the object recognition arena. The neuron was visually responsive (*F*_7,312_ > 10, *P* < 0.001), responding significantly (ANOVA: *F*_1,18_ = 5.34, *P* < 0.05) more strongly to novel than familiar stimuli. After visual stimulus offset, the neuron responded strongly (*F*_7,312_ > 10, *P* < 0.001) at the time of reward delivery (delivery of a food pellet within the arena, accompanied by an audible click). Rasters of action potential occurrences are shown above cumulated PSTHs for novel (first) and repeat (second) stimulus presentations. In the object arena, this neuron had activity that was significantly increased near objects (*F*_1,14_ = 7.98, *P* < 0.01) but produced no evidence of responding more to novel than familiar objects. B: The same neuron's increased activity near objects in the object recognition arena. (i) Plan view of arena: the dots represent the places where the cell fired during exploration of the arena. Two identical objects were located in the lower half of the arena as shown (“object zone”). (ii) Positional firing rate map of arena divided into 16 squares: activity was significantly increased in the “object zone” compared with the “no object zone” (*F*_1,14_ = 7.98, *P* < 0.01), a finding consistent with this cell having an “object field.” [Color figure can be viewed in the online issue, which is available at wileyonlinelibrary.com.]

The activity in squares adjacent to objects was then compared with activity in squares distant from objects both for Phase 1 and for Phase 2: Eight (53%) neurons (four from each of two rats) showed a significant change in mean activity between the squares near and not near objects in Phase 1 and/or Phase 2 (incidence, *P* < 0.001 compared with chance expectation). An example of such an increase in activity near objects is shown in Figure 7B. Of the eight changes, three were increases in activity near the objects and five decreases. Such changes in activity near to objects have been described as “object fields” (Burke et al., 2012; Deshmukh et al., 2012). As a control, activity in squares containing no objects was compared across the two phases; only one (7%) neuron changed activity significantly, an incidence close to that expected by chance (*P* > 0.1). When activity in squares near the three copies of object A in the two phases were compared with those near object B in Phase 2, again only one (7%) neuron showed significant change, providing no clear evidence that for these neurons the recorded activity changed with the identity of the object (*P* > 0.1 compared with chance). Analyses using smaller squares by dividing the arena into a 20 × 20 grid (each 4 × 4 cm, comparable to those analysed by Burke et al., 2012) proved unreliable because of false positives related to low spike counts, but nevertheless followed the same pattern as reported above, with putative “object fields” but no evidence of object or novelty discrimination.

When activity in the squares near novel objects in the two phases of the experiment (A in Phase 1 and B in Phase 2) was compared to activity near the familiar object (A) in Phase 2, no (0%) neuron showed a significant change. When activity in squares adjacent to the novel and familiar objects in Phase 2 were compared, for only one neuron (7%) was there a significant change; this neuron was not one of those with an object field, further suggesting it may have been a random change. In addition, no evidence of activity changes related to stimulus familiarity was found when the activity analysed was restricted to the first 4 min spent in the arena. These comparisons therefore provided no clear evidence of the encoding of information related to the relative familiarity of the object. In sum, activity changes were found near objects but it was not established that these changes encoded the identity of the object or its relative familiarity.

### Comparisons of Responsiveness in Experiments 1 and 2

The incidences of response changes related to stimulus familiarity were compared between the two different types of stimulus presentation in Experiments 1 and 2: 19 of 30 task responsive neurons changed response in the paired viewing arena and none of 8 in the object recognition arena, a significantly (Fisher Exact Probability Test, *P* < 0.01) lower incidence of changes related to relative familiarity in the object recognition arena. The perirhinal neuron whose responses are illustrated in Figure 7 was recorded in prescreening for the object recognition task and subsequently in the object recognition arena. This neuron responded more strongly to first than second presentations of initially novel pictures in prescreening; Figure 7A. However, when tested in the object recognition task, no difference was found in activity related to the relative familiarity of the objects, although there was evidence of significantly enhanced firing near objects (an “object field”); Figure 7B.

## DISCUSSION

The results confirm that a population of rat perirhinal neurons signal information concerning the relative familiarity of visual stimuli. When the stimuli are shown on a computer monitor in the ‘paired viewing’ arena the proportion of neurons responsive to stimulus familiarity was 16% of the total recorded, 30% of the visually responsive neurons. These figures are similar to those reported previously by (Zhu et al., 1995) and are in broad agreement with findings in the monkey (see for reviews, Brown and Xiang, 1998; Brown et al., 2010). They extend previous findings: (i) by establishing that all three patterns of response change for repeated presentations of novel and familiar stimuli that have been described in the monkey are also found in the rat and, (ii) by demonstrating that perirhinal neurons respond to 2‐D images shown on a computer screen (other work has used 3‐D objects). In contrast, when recordings were made in the object recognition arena no clear evidence was found for perirhinal neurons changing response in relation to the novelty or familiarity of an object. Perirhinal neuronal activity did change when the rat was near an object, providing evidence of “object fields” in agreement with previous findings (Burke et al., 2012; Deshmukh et al., 2012). Deshmukh et al. (2012) reported changes in firing near novel objects for 9% of a sample of 55 perirhinal neurons, but that proportion only approached being significantly above chance (*P* = 0.08). No evidence for the coding of relative familiarity was found by Burke et al. (2012), though their analysis involved a mean of first and second rather than solely first exposures. Moreover, the Burke et al. (2012) study used albino rats (Fisher 344 strain), which have lower visual acuity (Prusky et al., 2002) than the pigmented rats used here and by Deshmukh et al. (2012).

There are a number of possible reasons for the discordancy of findings related to the signalling of stimulus familiarity in the two recording situations investigated here in Experiments 1 and 2. First, the significance of a change in response between novel and familiar stimuli in the paired viewing arena was established by averaging across many (> 20) stimuli. In the object arena only two objects were used. Not all perirhinal neurons respond to all stimuli (Zhu and Brown, 1995; Burke et al., 2012; Deshmukh et al., 2012). Secondly, most of the responses in the paired viewing arena were relatively brief (<1 s). Moreover, for most differential neurons a strong response occurred only on the first viewing of a picture. Accordingly, a strong change in activity might only occur on the first viewing of an object (at the start of exploration or even when it is first caught sight of). In the object arena such a relatively fleeting change would probably be statistically undetectable in activity averaged across an exploration period of many seconds, particularly when the onset of stimulation (the time of the first sighting of the object) is not known. Thirdly, the picture presentations in the paired viewing arena (but not the object arena) were associated with reward (a drop of juice was delivered toward the end of stimulus display). In the object arena food pellet delivery was neither spatially nor temporally closely related to the object's presence. When a rat's attention is not engaged by such association of picture presentation with reward, perirhinal visual responses are difficult to find (M. Eldridge, R.U. Muller, and M.W. Brown, unpublished observations). Moreover, repetition‐related changes in neuronal responses were not found in monkey perirhinal cortex when monkeys passively viewed many stimuli where the familiarity of the stimuli was irrelevant to reward (Thome et al., 2012). Perirhinal neuronal responses have been shown to be sensitive to reward contingencies in the monkey (Liu and Richmond, 2000; Liu et al., 2000; Mogami and Tanaka, 2006) and monkey recordings are typically made in rewarded tasks. Moreover, early latency familiarity‐related MEG signals in the human are enhanced if performance of a task is rewarded (Bunzeck et al., 2009). Four other methodological issues may be discounted as reasons for the difference in findings. (i) In the paired viewing arena the stimuli were two‐dimensional, rather than three‐dimensional as in the object arena (Burke and Barnes, 2015). However, this difference is unlikely to be important as recognition‐related responsiveness has been found for 3‐D objects (Zhu and Brown, 1995; Zhu et al., 1995). (ii) The recording region sampled was similar in both situations – perirhinal cortex as defined by (Shi and Cassell, 1999) or posterior perirhinal cortex as defined by (Burwell, 2001). (iii) Although the rat strain differed for the two experiments, object fields have been found in both Long‐Evans (Deshmukh et al., 2012 and the present results) and Fisher 344 (Burke et al., 2012) strains. Moreover, recognition‐related neuronal responses have now been found in Long‐Evans (present results) as well as Dark Agouti (present and previous results) strains. The recording and analysis techniques, rat preparation, surgery, and handling, were all carried out by the same experimenters. Accordingly, it is unlikely that differences in the experimental results arise from differences in strain. (iv) The comparison between the two situations was partially biased in favour of finding visual responses in the object arena as there, but not for the paired viewing arena, neuronal populations were prescreened for visual responsiveness, but it was in the object arena that no evidence was found for activity changes related to familiarity.

There has been no systematic study of the encoding of visual stimulus identity by perirhinal neurons in the rat, although some evidence of apparent color selectivity has been reported (Zhu and Brown, 1995). “Object fields” are found when perirhinal neuronal activity is recorded as rats approach objects, but this technique is not well adapted to revealing what is being encoded concerning the identity of these objects: necessarily there are relatively few repetitions of a limited number of stimuli, with the added difficulty of determining stimulation onset time (plus the complicating factor that vision is not the only sense engaged). In contrast, in the monkey, using repeated presentations with known onset times, recording studies have established that detailed information concerning stimulus identity is encoded and transmitted by perirhinal neurons (Riches et al., 1991; Fahy et al., 1993; Sobotka and Ringo, 1993; Lueschow et al., 1994; Brown et al., 1996; Nakamura and Kubota, 1996; Erickson et al., 2000; Lehky and Tanaka, 2007; Fujimichi et al., 2010; Thome et al., 2012), findings in accord with the well‐established role of perirhinal cortex in perception (Buckley and Gaffan, 1998; Murray and Bussey, 1999; Bussey and Saksida, 2002; Murray et al., 2007).

There is overwhelming evidence from ablation and localised drug perfusion studies (see for reviews, (Dere et al., 2007; Winters et al., 2008; Brown et al., 2012; Brown and Banks, 2015) for the necessity of rat perirhinal cortex for recognition memory as measured in the standard object recognition memory task. Moreover, there are many reports of population measures of differences in perirhinal activity for novel and familiar stimuli (see for reviews, Brown et al., 2010, 2012; Aggleton et al., 2012; Brown and Banks, 2015). Moreover, structural equation modelling of counts of activated Fos neurons (Albasser et al., 2010) indicates that signals are passed from perirhinal cortex to the hippocampus concerning exploration of both novel and familiar objects during a serial recognition memory task based on spontaneous exploration. Accordingly, the failure to find perirhinal neuronal activity related to object familiarity in the object arena must concern its detectability by the measures used, rather than its total absence. Finding neuronal responses using individual neuron recording techniques carries the assumption that a not too small proportion of the sample will change activity by a not too small amount (otherwise the change will be statistically undetectable). It may be that detection of the activity change produced by a single novel object will need the use of arrays that can record simultaneously large a population of neurons, though it is likely also to require determination of when the object is first perceived by the rat. It is also possible that it may be necessary to make measurements of synchronous firing or oscillatory activity (Singer, 1993; Fries, 2008) in the object arena (although such measures were not needed to detect changes in the paired viewing arena). For the hippocampus, studying neuronal responsiveness by allowing a rat to repeatedly explore a static arena has been very successful (e.g., Muller, 1996); however, a parallel approach within sensory systems may not be optimal. In particular, within sensory systems efficiency of information processing is enhanced by signalling change rather than the constancy of stimulation across time and space: studying the neuronal signalling of sensory properties of an object by its constant presence may therefore be suboptimal. In these experiments such an approach failed to find signals related to object familiarity that were revealed by techniques more conventionally used in sensory neurophysiology.

## References

[hipo22584-bib-0001] Aggleton JP , Brown MW , Albasser MM. 2012 Contrasting brain activity patterns for item recognition memory and associative recognition memory: Insights from immediate‐early gene functional imaging. Neuropsychologia 50:3141–3155. 22634248 10.1016/j.neuropsychologia.2012.05.018

[hipo22584-bib-0002] Albasser M , Poirier G , Aggleton J. 2010 Qualitatively different modes of perirhinal‐hippocampal engagement when rats explore novel vs. familiar objects as revealed by c‐Fos imaging. Eur J Neurosci 31:134–147. 20092559 10.1111/j.1460-9568.2009.07042.x PMC4235254

[hipo22584-bib-0003] Barker GRI , Warburton EC. 2011 When is the hippocampus involved in recognition memory? J Neurosci 31:10721–10731. 21775615 10.1523/JNEUROSCI.6413-10.2011 PMC6622630

[hipo22584-bib-0004] Brown MW , Aggleton JP. 2001 Recognition memory: What are the roles of the perirhinal cortex and hippocampus? Nat Rev Neurosci 2:51–61. 11253359 10.1038/35049064

[hipo22584-bib-0005] Brown MW , Banks PJ. 2015 In search of a recognition memory engram. Neurosci Biobehav Rev 50:12–28. 25280908 10.1016/j.neubiorev.2014.09.016 PMC4382520

[hipo22584-bib-0006] Brown MW , Xiang JZ. 1998 Recognition memory: Neuronal substrates of the judgement of prior occurrence. Prog Neurobiol 55:149–189. 9618747 10.1016/s0301-0082(98)00002-1

[hipo22584-bib-0007] Brown MW , Wilson FA , Riches IP. 1987 Neuronal evidence that inferomedial temporal cortex is more important than hippocampus in certain processes underlying recognition memory. Brain Res 409:158–162. 3107754 10.1016/0006-8993(87)90753-0

[hipo22584-bib-0008] Brown MW , Fahy FL , Zhu XO. 1996 Studies of the recognition memory system In: Ono T, McNaughton BL, Molotchnikow S, Rolls ET, Nishijo H, editors. Perception, memory and emotion: frontiers in neuroscience. Amsterdam: Elsevier p 111–123.

[hipo22584-bib-0009] Brown MW , Warburton EC , Aggleton JP. 2010 Recognition memory: Material, processes, and substrates. Hippocampus 20:1228–1244. 20848602 10.1002/hipo.20858

[hipo22584-bib-0010] Brown MW , Barker GR , Aggleton JP , Warburton EC. 2012 What pharmacological interventions indicate concerning the role of the perirhinal cortex in recognition memory. Neuropsychologia 50:3122–3140. 22841990 10.1016/j.neuropsychologia.2012.07.034 PMC3500694

[hipo22584-bib-0011] Buckley MJ , Gaffan D. 1998 Perirhinal cortex ablation impairs visual object identification. J Neurosci 18:2268–2275. 9482811 10.1523/JNEUROSCI.18-06-02268.1998 PMC6792933

[hipo22584-bib-0012] Bunzeck N , Doeller CF , Fuentemilla L , Dolan RJ , Duzel E. 2009 Reward motivation accelerates the onset of neural novelty signals in humans to 85 milliseconds. Curr Biol 19:1294–1300. 19576774 10.1016/j.cub.2009.06.021 PMC2764383

[hipo22584-bib-0013] Burke SN , Barnes CA. 2015 The neural representation of 3‐dimensional objects in rodent memory circuits. Behav Brain Res 285:60–66. 25205370 10.1016/j.bbr.2014.09.001 PMC4362856

[hipo22584-bib-0014] Burke SN , Maurer AP , Hartzell AL , Nematollahi S , Uprety A , Wallace JL , Barnes CA. 2012 Representation of three‐dimensional objects by the rat perirhinal cortex. Hippocampus 22:2032–2044. 22987680 10.1002/hipo.22060 PMC3447635

[hipo22584-bib-0015] Burwell R. 2001 Borders and cytoarchitecture of the perirhinal and postrhinal cortices in the rat. J Comp Neurol 437:17–41. 11477594 10.1002/cne.1267

[hipo22584-bib-0016] Bussey T , Saksida L. 2002 The organization of visual object representations: A connectionist model of effects of lesions in perirhinal cortex. Eur J Neurosci 15:355–364. 11849301 10.1046/j.0953-816x.2001.01850.x

[hipo22584-bib-0017] Clark RE , Squire LR. 2013 Similarity in form and function of the hippocampus in rodents, monkeys, and humans. Proc Natl Acad Sci U S A 110:10365–10370. 23754372 10.1073/pnas.1301225110 PMC3690603

[hipo22584-bib-0018] Dere E , Huston JP , De Souza Silva MA. 2007 The pharmacology, neuroanatomy and neurogenetics of one‐trial object recognition in rodents. Neurosci Biobehav Rev 31:673–704. 17368764 10.1016/j.neubiorev.2007.01.005

[hipo22584-bib-0019] Deshmukh SS , Johnson JL , Knierim JJ. 2012 Perirhinal cortex represents nonspatial, but not spatial, information in rats foraging in the presence of objects: Comparison with lateral entorhinal cortex. Hippocampus 22:2045–2058. 22987681 10.1002/hipo.22046 PMC3870144

[hipo22584-bib-0020] Eichenbaum H , Yonelinas AP , Ranganath C. 2007 The medial temporal lobe and recognition memory. Annu Rev Neurosci 30:123–152. 17417939 10.1146/annurev.neuro.30.051606.094328 PMC2064941

[hipo22584-bib-0021] Ennaceur A , Delacour J. 1988 A new one‐trial test for neurobiological studies of memory in rats. 1: Behavioral data. Behav Brain Res 31:47–59. 3228475 10.1016/0166-4328(88)90157-x

[hipo22584-bib-0022] Erickson CA , Jagadeesh B , Desimone R. 2000 Clustering of perirhinal neurons with similar properties following visual experience in adult monkeys. Nat Neurosci 3:1143–1148. 11036272 10.1038/80664

[hipo22584-bib-0023] Eskandar EN , Richmond BJ , Optican LM. 1992 Role of inferior temporal neurons in visual memory. I. Temporal encoding of information about visual images, recalled images, and behavioral context. J Neurophysiol 68:1277–1295. 1432084 10.1152/jn.1992.68.4.1277

[hipo22584-bib-0024] Fahy FL , Riches IP , Brown MW. 1993 Neuronal activity related to visual recognition memory: Long‐term memory and the encoding of recency and familiarity information in the primate anterior and medial inferior temporal and rhinal cortex. Exp Brain Res 96:457–472. 8299747 10.1007/BF00234113

[hipo22584-bib-0025] Fries P. 2008 Neuronal gamma‐band synchronization as a fundamental process in cortical computation. Ann Rev Neurosci 32:209–224. 19400723 10.1146/annurev.neuro.051508.135603

[hipo22584-bib-0026] Fujimichi R , Naya Y , Koyano KW , Takeda M , Takeuchi D , Miyashita Y. 2010 Unitized representation of paired objects in area 35 of the macaque perirhinal cortex. Eur J Neurosci 32:659–667. 20718858 10.1111/j.1460-9568.2010.07320.x

[hipo22584-bib-0027] Gray CM , Maldonado PE , Wilson M , McNaughton B. 1995 Tetrodes markedly improve the reliability and yield of multiple single‐unit isolation from multi‐unit recordings in cat striate cortex. J Neurosci Methods 63:43–54. 8788047 10.1016/0165-0270(95)00085-2

[hipo22584-bib-0028] Huxter JR , Zinyuk LE , Roloff Ev L , Clarke VRJ , Dolman NP , More JCA , Jane DE , Collingridge GL , Muller RU. 2007 Inhibition of kainate receptors reduces the frequency of hippocampal theta oscillations. J Neurosci 27:2212–2223. 17329418 10.1523/JNEUROSCI.3954-06.2007 PMC6673475

[hipo22584-bib-0029] Kubie JL. 1984 A driveable bundle of microwires for collecting single‐unit data from freely‐moving rats. Physiol Behav 32:115–118. 6718521 10.1016/0031-9384(84)90080-5

[hipo22584-bib-0030] Lehky SR , Tanaka K. 2007 Enhancement of object representations in primate perirhinal cortex during a visual working‐memory task. J Neurophysiol 97:1298–1310. 17108097 10.1152/jn.00167.2006

[hipo22584-bib-0031] Li L , Miller EK , Desimone R. 1993 The representation of stimulus familiarity in anterior inferior temporal cortex. J Neurophysiol 69:1918–1929. 8350131 10.1152/jn.1993.69.6.1918

[hipo22584-bib-0032] Liu Z , Richmond BJ. 2000 Response differences in monkey TE and perirhinal cortex: Stimulus association related to reward schedules. J Neurophysiol 83:1677–1692. 10712488 10.1152/jn.2000.83.3.1677

[hipo22584-bib-0033] Liu Z , Murray EA , Richmond BJ. 2000 Learning motivational significance of visual cues for reward schedules requires rhinal cortex. Nat Neurosci 3:1307–1315. 11100152 10.1038/81841

[hipo22584-bib-0034] Lueschow A , Miller EK , Desimone R. 1994 Inferior temporal mechanisms for invariant object recognition. Cereb Cortex 4:523–531. 7833653 10.1093/cercor/4.5.523

[hipo22584-bib-0035] Miller EK , Desimone R. 1994 Parallel neuronal mechanisms for short‐term memory. Science 263:520–522. 8290960 10.1126/science.8290960

[hipo22584-bib-0036] Miller EK , Li L , Desimone R. 1993 Activity of neurons in anterior inferior temporal cortex during a short‐term memory task. J Neurosci 13:1460–1478. 8463829 10.1523/JNEUROSCI.13-04-01460.1993 PMC6576733

[hipo22584-bib-0037] Mogami T , Tanaka K. 2006 Reward association affects neuronal responses to visual stimuli in macaque te and perirhinal cortices. J Neurosci 26:6761–6770. 16793883 10.1523/JNEUROSCI.4924-05.2006 PMC6673829

[hipo22584-bib-0038] Muller R. 1996 A quarter of a century of place cells. Neuron 17:813–822. 8938115 10.1016/s0896-6273(00)80214-7

[hipo22584-bib-0039] Muller RU , Kubie JL , Ranck JB Jr . 1987 Spatial firing patterns of hippocampal complex‐spike cells in a fixed environment. J Neurosci 7:1935–1950. 3612225 10.1523/JNEUROSCI.07-07-01935.1987 PMC6568929

[hipo22584-bib-0040] Murray E , Bussey T. 1999 Perceptual‐mnemonic functions of the perirhinal cortex. Trends Cogn Sci 3:142–151. 10322468 10.1016/s1364-6613(99)01303-0

[hipo22584-bib-0041] Murray E , Bussey T , Saksida L. 2007 Visual perception and memory: A new view of medial temporal lobe function in primates and rodents. Annu Rev Neurosci 30:99–122. 17417938 10.1146/annurev.neuro.29.051605.113046

[hipo22584-bib-0042] Nakamura K , Kubota K. 1996 The primate temporal pole: Its putative role in object recognition and memory. Behav Brain Res 77:53‐ 77: 8762159 10.1016/0166-4328(95)00227-8

[hipo22584-bib-0043] Paxinos G , Watson C. 2005. The rat brain in stereotaxic coordinates. Amsterdam; Boston: Elsevier Academic Press.

[hipo22584-bib-0044] Prusky GT , Harker KT , Douglas RM , Whishaw IQ. 2002 Variation in visual acuity within pigmented, and between pigmented and albino rat strains. Behav Brain Res 136:339–348. 10.1016/s0166-4328(02)00126-2 12429395

[hipo22584-bib-0045] Ranganath C , Ritchey M. 2012 Two cortical systems for memory‐guided behaviour. Nat Rev Neurosci 13:713–726. 22992647 10.1038/nrn3338

[hipo22584-bib-0046] Riches I , Wilson F , Brown M. 1991 The effects of visual stimulation and memory on neurons of the hippocampal formation and the neighboring parahippocampal gyrus and inferior temporal cortex of the primate. J Neurosci 11:1763–1779. 2045886 10.1523/JNEUROSCI.11-06-01763.1991 PMC6575394

[hipo22584-bib-0047] Ringo JL. 1996 Stimulus specific adaptation in inferior temporal and medial temporal cortex of the monkey. Behav Brain Res 76:191–197. 8734053 10.1016/0166-4328(95)00197-2

[hipo22584-bib-0048] Shi C , Cassell M. 1999 Perirhinal cortex projections to the amygdaloid complex and hippocampal formation in the rat. J Comp Neurol 406:299–328. 10102498 10.1002/(sici)1096-9861(19990412)406:3<299::aid-cne2>3.0.co;2-9

[hipo22584-bib-0049] Singer W. 1993 Synchronization of cortical activity and its putative role in information processing and learning. Ann Review Phys 55:349–374. 10.1146/annurev.ph.55.030193.002025 8466179

[hipo22584-bib-0050] Sobotka S , Ringo J. 1993 Investigation of long term recognition and association memory in unit responses from inferotemporal cortex. Exp Brain Res 96:28–38. 8243581 10.1007/BF00230436

[hipo22584-bib-0051] Sobotka S , Ringo JL. 1996 Mnemonic responses of single units recorded from monkey inferotemporal cortex, accessed via transcommissural versus direct pathways: A dissociation between unit activity and behavior. J Neurosci 16:4222–4230. 8753883 10.1523/JNEUROSCI.16-13-04222.1996 PMC6579006

[hipo22584-bib-0052] Thome A , Erickson CA , Lipa P , Barnes CA. 2012 Differential effects of experience on tuning properties of macaque MTL neurons in a passive viewing task. Hippocampus 22:2000–2011. 22987678 10.1002/hipo.22070 PMC3537226

[hipo22584-bib-0053] Wan H , Aggleton JP , Brown MW. 1999 Different contributions of the hippocampus and perirhinal cortex to recognition memory. J Neurosci 19:1142–1148. 9920675 10.1523/JNEUROSCI.19-03-01142.1999 PMC6782155

[hipo22584-bib-0054] Warburton EC , Koder T , Cho K , Massey PV , Duguid G , Barker GRI , Aggleton JP , Bashir ZI , Brown MW. 2003 Cholinergic neurotransmission is essential for perirhinal cortical plasticity and recognition memory. Neuron 38:987–996. 12818183 10.1016/s0896-6273(03)00358-1

[hipo22584-bib-0055] Warburton EC , Glover CPJ , Massey PV , Wan H , Johnson B , Bienemann A , Deuschle U , Kew JNC , Aggleton JP , Bashir ZI , Uney J . 2005 cAMP responsive element‐binding protein phosphorylation is necessary for perirhinal long‐term potentiation and recognition memory. J Neurosci 25:6296–6303. 16000619 10.1523/JNEUROSCI.0506-05.2005 PMC6725268

[hipo22584-bib-0056] Winters BD , Saksida LM , Bussey TJ. 2008 Object recognition memory: Neurobiological mechanisms of encoding, consolidation and retrieval. Neurosci Biobehav Rev 32:1055–1070. 18499253 10.1016/j.neubiorev.2008.04.004

[hipo22584-bib-0057] Xiang JZ , Brown MW. 1998 Differential neuronal encoding of novelty, familiarity and recency in regions of the anterior temporal lobe. Neuropharmacol 37:657–676. 10.1016/s0028-3908(98)00030-6 9705004

[hipo22584-bib-0058] Young BJ , Otto T , Fox GD , Eichenbaum H. 1997 Memory representation within the parahippocampal region. J Neurosci 17:5183–5195. 9185556 10.1523/JNEUROSCI.17-13-05183.1997 PMC6573311

[hipo22584-bib-0059] Zhu XO , Brown MW. 1995 Changes in neuronal activity related to the repetition and relative familiarity of visual stimuli in rhinal and adjacent cortex of the anaesthetised rat. Brain Res 689:101–110. 8528693 10.1016/0006-8993(95)00550-a

[hipo22584-bib-0060] Zhu XO , Brown MW , Aggleton JP. 1995 Neuronal signalling of information important to visual recognition memory in rat rhinal and neighbouring cortices. Eur J Neurosci 7:753–765. 7620624 10.1111/j.1460-9568.1995.tb00679.x

[hipo22584-bib-0061] Zhu XO , McCabe BJ , Aggleton JP , Brown MW. 1996 Mapping visual recognition memory through expression of the immediate early gene c‐fos. Neuroreport 7:1871–1875. 8905683 10.1097/00001756-199607290-00037

[hipo22584-bib-0062] Zynyuk L , Huxter J , Muller RU , Fox SE. 2012 The presence of a second rat has only subtle effects on the location‐specific firing of hippocampal place cells. Hippocampus 22:1405–1416. 21997883 10.1002/hipo.20977

